# Bilateral Renal Agenesis/Hypoplasia/Dysplasia (BRAHD): Postmortem Analysis of 45 Cases with Breakpoint Mapping of Two *De Novo* Translocations

**DOI:** 10.1371/journal.pone.0012375

**Published:** 2010-08-25

**Authors:** Louise Harewood, Monica Liu, Jean Keeling, Alan Howatson, Margo Whiteford, Peter Branney, Margaret Evans, Judy Fantes, David R. FitzPatrick

**Affiliations:** 1 MRC Human Genetics Unit, Institute of Genetic and Molecular Medicine, Edinburgh, United Kingdom; 2 Medical School, University of Edinburgh, Edinburgh, United Kingdom; 3 Department of Paediatric Pathology, New Royal Infirmary, Edinburgh, United Kingdom; 4 Department of Paediatric Pathology, Royal Hospital for Sick Children, Glasgow, United Kingdom; 5 Department of Clinical Genetics, Royal Hospital for Sick Children, Glasgow, United Kingdom; 6 South-East Scotland Regional Genetics Services, Western General Hospital, Edinburgh, United Kingdom; Leiden University Medical Center, Netherlands

## Abstract

**Background:**

Bilateral renal agenesis/hypoplasia/dysplasia (BRAHD) is a relatively common, lethal malformation in humans. Established clinical risk factors include maternal insulin dependent diabetes mellitus and male sex of the fetus. In the majority of cases, no specific etiology can be established, although teratogenic, syndromal and single gene causes can be assigned to some cases.

**Methodology/Principal Findings:**

45 unrelated fetuses, stillbirths or infants with lethal BRAHD were ascertained through a single regional paediatric pathology service (male∶female 34∶11 or 3.1∶1). The previously reported phenotypic overlaps with VACTERL, caudal dysgenesis, hemifacial microsomia and Müllerian defects were confirmed. A new finding is that 16/45 (35.6%; m∶f 13∶3 or 4.3∶1) BRAHD cases had one or more extrarenal malformations indicative of a disoder of laterality determination including; incomplete lobulation of right lung (seven cases), malrotation of the gut (seven cases) and persistence of the left superior vena cava (five cases). One such case with multiple laterality defects and sirelomelia was found to have a *de novo* apparently balanced reciprocal translocation 46,XY,t(2;6)(p22.3;q12). Translocation breakpoint mapping was performed by interphase fluorescent *in-situ* hybridization (FISH) using nuclei extracted from archival tissue sections in both this case and an isolated bilateral renal agenesis case associated with a *de novo* 46,XY,t(1;2)(q41;p25.3). Both t(2;6) breakpoints mapped to gene-free regions with no strong evidence of *cis*-regulatory potential. Ten genes localized within 500 kb of the t(1;2) breakpoints. Wholemount *in-situ* expression analyses of the mouse orthologs of these genes in embryonic mouse kidneys showed strong expression of *Esrrg*, encoding a nuclear steroid hormone receptor. Immunohistochemical analysis showed that Esrrg was restricted to proximal ductal tissue within the embryonic kidney.

**Conclusions/Significance:**

The previously unreported association of BRAHD with laterality defects suggests that renal agenesis may share a common etiology with heterotaxy in some cases. Translocation breakpoint mapping identified *ESRRG* as a plausible candidate gene for BRAHD.

## Introduction

Bilateral renal agenesis/hypoplasia/dysplasia (BRAHD) is collective term used here to describe a group of lethal renal malformations. The EUROCAT registry (a European network of population-based registries for the epidemiologic surveillance of congenital anomalies) reports an incidence of 1.3 per 10,000 live births, fetal deaths/still births and terminations of pregnancy for fetal anomaly (http://www.eurocat.ulster.ac.uk/pdf/EUROCAT-Final-EC-Report.pdf). BRAHD shows a male preponderance with a male∶female ratio of 2.5∶1. Potter syndrome describes the constellation of extrarenal clinical features commonly associated with BRAHD; wide-set eyes, 'squashed' nose, receding chin, large, low-set ears deficient in cartilage, deformity of the feet and hands and hypoplasia of the lungs [1], [2]. Potter syndrome is considered to be the paradigm for a birth defect “sequence” in which a primary malformation results in a series of secondary birth defects. In the case of BRAHD, the primary malformation is an intrinsic error of renal development with the other features of Potter syndrome resulting from intrauterine deformation due to oligohydramnios. It should be noted, however, that Potter syndrome may also be the result of obstruction of urinary excretion caused by a primary abnormality of the lower urinary tract [3].

Kidney development in vertebrates begins with the formation of the primary nephric duct - symmetric bilateral cords of epithelial cells derived from the intermediate mesoderm [4]. In humans, the nephric duct appears at 22 gestational days (GD). Between 28 and −56 GD, there is transient formation of a functional embryonic kidney, the mesonephros, which is structurally similar to the glomerular and collecting systems found in fish [5]. In males the portion of the nephric duct that drains the mesonephros will form the epididymis, seminiferous vesicle and vas deferens in post-embryonic life. The fallopian tubes, uterus and upper vagina - the Müllerian structures - develop from paramesonephic tissue in female embryos [6]. Finally, the definitive kidney or metanephros is formed when the ureteric bud, an outgrowth of the distal nephric duct, undergoes extensive branching and induces the surrounding mesoderm to form glomeruli and nephrons [7], [8].

Genetic factors are clearly an important component in the etiology of BRAHD. There is significant familial aggregation of cases with an empiric sibling recurrence risk for BRAHD of approximately 5% [9]–[12]. Parents or siblings of infants with BRAHD have an increased incidence of less severe kidney malformations such as renal agenesis and/or renal dysplasia that may be bilateral or unilateral, suggesting an autosomal dominant condition with incomplete penetrance and variable expressivity [13]–[15]. Complete non-penetrance has also been reported [9]. 148 different syndrome entities associated with renal agenesis are listed in the Winter-Barraitser Dysmorphology Database (London Medical Databases, London). The most commonly reported BRAHD-associated single gene disorders are Fraser syndrome (OMIM 219000) [16], [17] and Branchio-Oto-Renal syndrome (BOR, OMIM 113650) [18].

Recently, mutations in *RET* have been identified in 7/19 (36.8%) of cases in a cohort of BRAHD [19]. Eight different mutations were identified in these seven BRAHD cases. Surprisingly both activating and inactivating mutations in *RET* were identified but no convincing mutations were found in the genes encoding a co-receptor of RET, *GFRA*, or their ligand, *GDNF*. These genes became strong candidates for BRAHD following the identification of renal agenesis as a prominent feature in mouse embryos with targeted inactivation of the loci [20]–[22]. A missense mutation in the paired domain of PAX2 has been identified in a single, large autosomal dominant family with renal dysplasia as an isolated malformation showing highly variable expression [23]. This is interesting because heterozygous, loss-of-function mutations in the gene encoding PAX2 have been associated most commonly associated with renal-coloboma syndrome (OMIM 120330).

In addition to genetic factors, there is a strong association between BRAHD and maternal insulin-dependent diabetes mellitus (IDDM) [24]. This appears be the result of a direct teratogenic effect of hyperglycaemia on specific tissues within the embryo [25].

In this paper we report detailed phenotypic characterization of 45 cases of BRAHD with particular reference to the associated non-renal malformations. We identify a strong and previously unreported association with heterotaxy. We also report interphase fluorescent *in-situ* hybridization (FISH) mapping of two *de novo* apparently balanced chromosomal rearrangements associated with BRAHD and determine the embryonic kidney expression of genes positioned close to the breakpoints. *Esrrg* showed the strongest expression during renal development in the mouse and may represent a candidate gene for future functional and genetic studies.

## Methods

### Case ascertainment

Cases were ascertained by searches of the fetal and perinatal post-mortem (PM) reports generated by a single regional pediatric pathology service over an eleven-year period between 1993–2003. This work was carried out in a manner consistent with ethical principles for medical research involving human subjects outlined in the Declaration of Helsinki and with the approval of the Lothian Regional Ethics Committee (REF:2000/6/53). All families had given written consent for research use of tissue and clinical information at the time of post-mortem (PM) examination. The PM records were stored in a custom-designed 4D database (4D, San Jose, USA) using a structured vocabulary for coding clinical features. All reports with evidence of bilateral renal malformations or a diagnosis of Potter syndrome were reviewed in detail. The term ‘agenesis’ was used when no kidney tissue could be identified on autopsy, and the term ‘dysplasia’ was used when abnormal kidney structure was described on autopsy or histology e.g. cystic dysplasia. ‘Renal hypoplasia’ was used to describe a significantly small kidney without cystic dysplasia. Information on maternal age and parity, prenatal/birth history and associated malformations was recorded. Reliable family history information was generally unobtainable from the PM reports and most parents had not been referred to clinical genetics services. The criteria for inclusion in this study were lethal bilateral renal agnesis OR renal hypoplasia OR renal dysplasia. Infants with unilateral renal malformation with a normal contralateral kidney were excluded. Cytogenetic reports were sought for each of the cases identified with BRAHD in the searches detailed above using the computerized archive of the clinical cytogenetic laboratory.

### Interphase FISH Mapping

Interphase FISH mapping on nuclei isolated from clinically archived formalin-fixed, paraffin-embedded (FFPE) tissue sections was performed as previously reported [26]. Briefly, 10 µm sections were dissociated in pepsin (4 mg/ml in 10 mM HCl at 37°C) for four hours, filtered using a 40 µm cell strainer (Falcon) and rinsed through with PBS, before being fixed in 3∶1 methanol∶acetic acid fix. BACs, PACs and fosmids were obtained from BACPAC Resource Center (BPRC) at Children's Hospital Oakland Research Institute, or the Wellcome Trust Sanger Institute, Cambridge. Clones were grown and DNA extracted according to BPRC protocol. A full list of the clones used to map both breakpoints is available in Table S2. FISH probes were produced and FISH performed, according to standard methods, on fixed cell suspensions [27] or on FFPE isolated nuclei suspensions (Harewood, 2010). Chromosome paints were a kind gift from Dr Jeff Trent [28].

Interphase FISH mapping of reciprocal translocations requires the examination of multiple (at least five fully interpretable) nuclei for each probe to determine consistent co-localization or failure of co-localization of co-hybridized pairs of BAC probes, or one BAC probe and a chromosome paint (labeled with different fluorochromes). In reciprocal translocations the presence of a normal allele in each nucleus provides a useful control. This approach has been successfully used to map disease-related reciprocal translocations in both peripheral blood leukocyte nuclei [29] and FFPE tissue [26].

### Expression analysis in mouse embryonic kidneys

Wholemount *in-situ* hybridization (WISH) was performed on kidneys dissected from 14.5dpc wild-type mouse embryos. PCR of mouse genomic DNA was used to generate riboprobe DNA templates for the 1p and 2p breakpoint genes and digoxigenin (DIG, Roche)-labeled antisense riboprobes generated by *in vitro* transcription using T7 RNA polymerase. Embryonic kidneys were washed in PBST (PBS + 0.1% Tween 20) and permeabilised with Proteinase K (10 µg/ml) for 20 minutes, then washed twice in 0.1 M triethanolamine, with the addition of acetic anhydride to the second wash. The kidneys were re-fixed in 4% PFA/0.2% gluteraldehyde for 20 minutes and washed extensively in PBST. The kidneys were then placed in 2 ml tubes and prehybridized in hybridization buffer for 2 hours in a 60°C shaking water bath before being hybridized for 2 nights at 60°C in fresh hybridization buffer containing the DIG-labeled probe. Probe solution was removed and the kidneys washed twice in fresh hybridization buffer, at 60°C, for ten minutes. Samples were then washed three times in 2x SSC + 0.1% Tween 20 for 20 minutes per wash and three times in 0.2x SSC + 0.1% Tween 20 for 30 minutes per wash (all at 60°C) before being washed twice in maleic acid buffer (MAB) for 15 minutes per wash at room temperature. MAB was replaced with a MAB + 2% BMB (Boehringer-Mannheim blocking reagent) + 20% heat-treated lamb serum solution and kidneys were left for two hours at room temperature with gentle agitation. After two hours, a 1/2000 dilution of anti-DIG antibody coupled to alkaline phosphatase (Roche) in the same solution was added and left overnight at 4°C, then washed three times in MAB (5 min per wash) and three times in MAB for one hour per wash. Color detection was performed using 2 ml of BM purple precipitating solution (Roche).

Immunohistochemical localization of ESRRG was performed using a rabbit polyclonal antisera (AbCam, cat ab12988) on sections of paraffin embedded wild type CD1 mouse 14.5 dpc embryos at a dilution of 1 in 500. 4–6 µm sections were de-waxed, rehydrated and microwaved in boiling 10 mM citrate buffer twice for 30 seconds and left to cool in the buffer for 20 minutes. Heat-inactivated sheep serum as a 10% solution in PBS was applied for at least one hour, at room temperarure, to reduce non-specific binding. The slides were incubated in the same solution, with the addition of the primary antibody, overnight in a humidified chamber at 4°C, washed in PBS and PBST (five minutes each) and the secondary antibody (biotinylated anti-rabbit IgG, 1 in 1000) applied for one hour at room temperature before being washed as before. Detection was performed using the Vector Lab ABC kit with NBT/BCIP and sections were counterstained with eosin.

## Results

### Basic Clinical Data

45 cases of bilateral renal agenesis and/or hypoplasia and/or dysgenesis (BRAHD) were identified from postmortem records: 20 cases (44.4%) had bilateral renal agenesis, 15 (33.3%) had unilateral renal agenesis (URA) with either hypoplasia or dysplasia affecting the contralateral kidney, and 10 cases (22.2%) had bilateral renal dysplasia. In the 15 URA cases, the right kidney was absent in ten cases and the left kidney absent in five cases. Of the bilateral dysplastic group there were three specific histological diagnoses; two cases with bilateral medullary dysplasia (one of these had Meckel Gruber syndrome) and one case with bilateral renal tubular dysplasia (this case had trisomy 13). The male∶female ratio in the complete BRAHD cohort is 34∶11 (3.1∶1). In the cases with either unilateral or bilateral renal agenesis the ratio is 29∶6 (4.8∶1). In the ten cases with bilateral renal dyplasia there was an equal sex distribution (5∶5). As expected, oligohydramios-associated deformations were common - characteristic “Potter facies” were reported in 38/45 (84.4%) cases, lung hypoplasia in 23/45 (51.1%) and talipes equinovarus in 25/45 (55.6%).

### Associated Non-renal Malformations

31/45 (68.9%) cases had one or more extrarenal malformations (male∶female ratio 2.44∶1). There were 175 extrarenal malformations recorded in these cases consisting of 80 different malformation types. A full list of the associated malformations is given in Table S1. 27 different extrarenal malformations were identified in two or more cases (Table 1). The most common categories of major malformations were: GI tract atresias (13 cases of anorectal atresia, four cases of esophageal atresia, two cases of colonic atresia and one case of multiple small bowel atresia) and axial skeletal malformations (seven cases of thoracic vertebral malformation, three cases with an abnormal number of pairs of ribs, two cases of lumbosacral vertebral malformations, two cases of sacral agenesis and one case of cervical rachischisis). A definitive autosomal recessive syndromal diagnosis could be made on clinical grounds in three cases; Meckel Gruber syndrome (OMIM 249000), Renal-Hepatic-Pancreatic Dysplasia syndrome (OMIM 208540) and Fraser syndrome (OMIM 219000) (Table 2).

**Table 1:** Extrarenal malformations seen in more than one case.

Malformation	Total
Undescended testes	15
Anorectal Atresia	13
Cervical/thoracic vertebral malformation	9
Incomplete lobulation right lung	7
Malrotation of gut	7
Radial aplasia	7
Cleft palate	5
Micrognathia	5
Cerebellar hypoplasia	4
Cleft lip	4
Meckel Diverticulum	4
Esophageal atresia	4
Persistent left superior vena cava	5
Ventricular septal defect (VSD)	4
Bicornuate uterus	3
Transposition of the great arteries (TGA)	3
Tracheo-Esophageal fistula	3
Abnormal number of pairs of ribs	3
Urethral atresia/obstruction	3
Absent penis	2
Colonic atresia	2
Ductal Plate Malformation	2
Lumbosacral vertebral malformations	2
Malformation of olivary nucleus	2
Postaxial polydactyly of upper limbs	2
Preaxial polydactyly of lower limbs	2
Rectovesicular fistula	2

**Table 2:** Analysis of Reported Association in Cases with Extrarenal Malformations.

PM Number	Sex	Left Kidney	Right Kidney	V	A	C	TE	R	L	VACTERL Score	Mü	CD	HFM	Lat	Mat IDDM	Cat	Syndrome
83	F	Hypoplastic	Agenesis	Y		Y		Y	Y	4				C			
122	M	Agenesis	Agenesis	Y		Y	Y	Y	Y	5			Y	CR			
3029	M	Cystic Dysplasia	Agenesis	Y	Y	Y		Y	Y	5				AC			
3081	M	Cystic Dysplasia	Agenesis	Y	Y	Y		Y	Y	5				AC		Chr	Mosaic trisomy 8
3133	M	Agenesis	Agenesis			Y		Y		2							
3177	M	Agenesis	Agenesis					Y		1							
94189	M	Agenesis	Agenesis					Y		1		Y		R			
94271	F	Cystic Dysplasia	Hypoplastic			Y		Y		2							
95050	M	Hypoplastic	Hypoplastic and Dysplastic					Y		1							
95054	F	Agenesis	Agenesis					Y		1	Y						
95076	M	Agenesis	Agenesis		Y	Y		Y	Y	4		Y		RC			
95311	M	Agenesis	Hypoplastic					Y		1						AR	Fraser Syndrome
95363	M	Cystic Dysplasia	Agenesis					Y		1							
95369	M	Cystic Dysplasia	Hypoplastic					Y		1							
96080	M	Cystic Dysplasia	Agenesis	Y	Y	Y		Y	Y	5		Y	Y	ARC	Y	Chr	t(2;6)(p23;q14) dn
96107	M	Cystic Dysplasia	Agenesis		Y	Y		Y		3		Y		ARC			
96218	M	Cystic Dysplasia	Agenesis	Y	Y			Y		3		Y		A	Y		
96221	F	Dysplastic	Dysplastic					Y		1						Chr	Trisomy 13
96431	M	Dysplastic	Hypoplastic & Cystic Dysplasia	Y	Y	Y	Y	Y		5		Y		C	Y		
96489	M	Agenesis	Cystic Dysplasia	Y		Y		Y		3							
97019	F	Agenesis	Agenesis					Y		1	Y						
97099	M	Agenesis	Agenesis	Y	Y			Y	Y	4		Y					
97219	M	Cystic Dysplasia	Cystic Dysplasia	Y	Y		Y	Y		4		Y		C			
97262	F	Agenesis	Agenesis					Y		1				R			
98045	M	Agenesis	Agenesis		Y			Y		2		Y		A			
98074	F	Hypoplastic & Cystic Dysplasia	Hypoplastic & Cystic Dysplasia					Y		1							
98223	F	Agenesis	Agenesis	Y	Y			Y		3	Y	Y		A			
98305	M	Cystic Dysplasia	Agenesis		Y	Y		Y	Y	4			Y	C			
98307	M	Agenesis	Agenesis		Y	Y		Y		3				RC			
99069	F	Cystic Dysplasia	Cystic Dysplasia					Y		1						AR	Meckel Gruber Syndrome
99143	M	Agenesis	Agenesis					Y		1						AR	Renal-Hepatic-Pancreatic Dysplasia syndrome

Abbreviations: V – Vertebral malformations, A – Anal atresia, C – Cardiovascular anomalies, TE – Tracheo-esophogeal fistula/Esophogeal atresia, R – Renal anomalies, L – Limb malformations, Mü – Müllerian duct anomalies, CD - Caudal dysgenesis, HFM – Hemifacial microsomia, Lat - Defect in Laterality Determination A - abdominal laterality defect, R - respiratarory tract laterality defect, C - cardiac laterality defecr Mat IDDM – Maternal insulin-dependent diabetes mellitus, Cat – Category: Chr – Chromosomal abnormality, AR – Autosomal recessive.

The cases with extrarenal malformations were further characterized by whether they had any component of three well known malformation associations (Table 2) considered to overlap with BRAHD, namely VACTERL association (vertebral malformation, anal atresia, cardiovascular anomalies, tracheo-esophageal fistula, esophageal atresia, renal anomalies, limb malformations [OMIM 192350]) [30], Müllerian duct anomalies (MURCS association [OMIM 601076], Mayer-Rokitansky-Kuster-Hauser Syndrome [OMIM 277000]) [31], [32] or hemifacial microsomia (Goldenhar syndrome/Oculo-auriculo-vertebral syndrome [OMIM 164210]) [33], [34]. Excluding the renal malformations for which the cases were selected, 18/31 (58.1%) cases had at least one other feature consistent with VACTERL association (and hence a VACTERL score of 2), 15/31 (48.4%) had two or more other features (VACTERL score of 3), 10/31 (32.2%) had three or more (VACTERL 4) and 5/31 (16.1%) had four other components of VACTERL. Three of the nine female BRAHD cases with extrarenal malformations had Müllerian duct anomalies and 3/31 (9.7%) cases had hemifacial microsomia.

Partial laterality defects were identified in 16/31 (51.6%) cases. The most common manifestation of heterotaxy was incomplete lobulation of right lung (seven cases), malrotation of the gut (seven cases), and persistence of the left superior vena cava (five cases) (Table 2).

### Cytogenetic Analysis and Clinical Details of Translocation Cases

**Figure 1:**
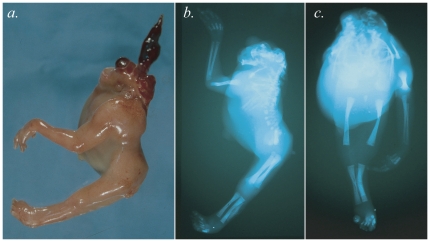
Clinical and radiological features of t(2;6) case. A. shows a left lateral photograph of this fetus with craniorachischisis and severely abnormal facial structures. B. Lateral radiograph showing the left upper limb with mild radial aplasia and the absence of the right upper limb. The axial skeleton is severely abnormal with multiple thoracocostal anomalies. C. AP radiograph showing sirenomelia and extensive axial skeletal malformations.

Chromosome studies were obtained in 32 of the 45 cases. In 3/32 (9.4%) of the cases the chromosomal analysis was reported as abnormal. Two numerical chromosome abnormalities were identified: PM number 96221 with 46,XX,+13 and 3081 with 47,XY,+8[2]/46XY[28]. One structural chromosomal anomaly was detected - a *de novo*, apparently balanced reciprocal translocation, 46,XY,t(2;6)(?p23;?q14), in a pregnancy that was terminated at 18 weeks of gestation following an ultrasound diagnosis of anencephaly with multiple other congenital anomalies. The mother had insulin-dependent diabetes mellitus. On autopsy, this fetus had craniorachischisis extending to the upper thoracic region (Figure 1). There was marked facial asymmetry associated with right-sided anophthalmia and a wide “irregular” cleft of the upper lip. The left upper limb was present and had mild radial aplasia. The right upper limb was absent with a small digit attached to the upper thorax. There was sirenomelia associated with anal atresia and malformed genital tubercle. There was a single umbilical artery. Internal examination revealed a normal stomach, duodenum, pancreas and spleen. The lungs had undergone only rudimentary lobulation. The right kidney was absent and the left kidney had cystic dysplasia. The ureters were narrow and inserted into the blind ending bowel. There was transposition of the great arteries and a ventricular septal defect.

In the course of this study we identified another case of BRAHD associated with a *de novo*, apparently balanced reciprocal translocation. Details of the clinical and cytogenetic features of this case have been reported elsewhere [35] although no molecular mapping of the breakpoints had previously been carried out. Briefly, this was the first pregnancy of a healthy and non-consanguineous couple. At 29 weeks, oligohydramnios was noted and an anomaly scan was suggestive of bilateral renal agenesis. At 32 weeks, lung hypoplasia was apparent and the parents opted to have labor induced. The baby died an hour after birth. On PM examination the baby was male and had features of Potter sequence, including a flattened nose, large squashed ears, rocker bottom feet and marked skin laxity over the trunk and limbs. Hemorrhagic masses with no recognizable renal structuring were found in the place of kidneys. Histology revealed undifferentiated mesenchyme with foci of cartilage. Cytogenetic analysis identified an apparently balanced reciprocal translocation, 46,XY,t(1;2)(q32;p25) which had occurred *de novo*.

### Interphase FISH Mapping

**Figure 2:**
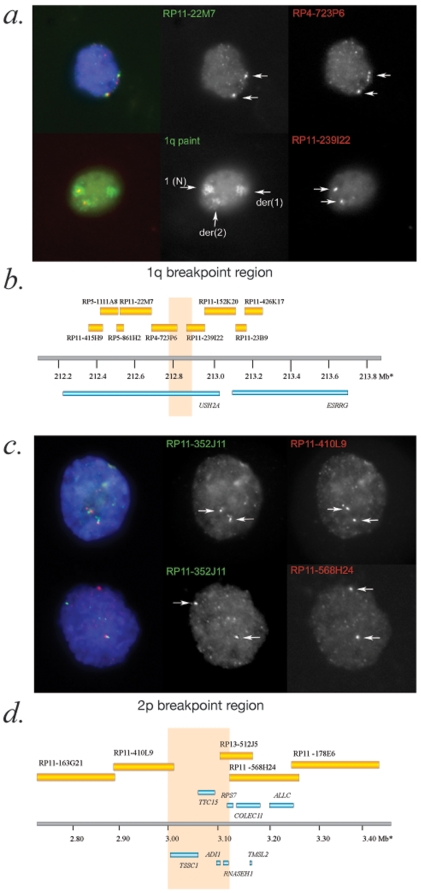
Mapping of the t(1;2) breakpoints using interphase FISH analysis. As no metaphase spreads or viable cells were available from this case, interphase mapping was performed using dissociated nuclei from archival tissue sections. The mapping of each clone was confirmed on multiple nuclei to confirm the presence or absence of co-localization. A. Two representative nuclei used to map probes flanking the 1q breakpoint. On the left is the merged color image with the green and red channels of the same image shown in the middle and the right-hand-side respectively. The top nucleus shows co-localization of the FISH signals for RP11-22M7 and RP4-723P6 on both chromosomes (white arrows), which together with other results shown in Table S2, can be used to map the more telomeric probe (RP4-723P6) to the der(1). The lower nucleus demonstrates the utility of chromosome arm paints in interphase mapping. The green channel image shows three clear 1q chromosomal paint domains within the nucleus. On the basis of the relative volume of these domains and with knowledge of the cytogenetic location of the translocation breakpoint, it is possible to assign these domains to the normal chromosome 1 (1(N), the largest domain) and the derivative chromosomes (der(1), middle-sized domain and der(2), smallest domain). It can be seen that the RP11-239I22 probe maps to the der(2) and is therefore distal to the 1q breakpoint. B. The physical map of the breakpoint region of 1q with the orange box indicating the region of genomic DNA that could plausibly contain the breakpoint. There are only two genes in this region with one of these, *USH2A*, being directly disrupted by the breakpoint. C. Two representative nuclei used in mapping the 2p breakpoint. The overall configuration is identical to A. The upper nucleus shows colocalization of RP11-410L9 with a probe (RP11-352J11) that has been shown to map telomeric of the 2p breakpoint. The lower nucleus shows failure of colocalization of RP11-568H24 on one allele, showing that this maps to the der(2). D. The physical map of the breakpoint region (orange box) on 2p. Unfortunately, it was not possible to map this breakpoint further. Five genes could plausibly be disrupted by this breakpoint; *TSSC1*, *TTC15*, *ADI1*, *RNASEH1* and *RPS7*.

Unfortunately, no metaphase chromosome preparations or viable cell-lines were available from either of the *de novo* translocation cases. We therefore proceeded to map both translocations using interphase nuclei from paraffin embedded tissue by observing the presence or absence of co-localization of pairs of fluorescently labeled BAC probes or chromosome paints. Mapping of the 46,XY,t(1;2)(q32;p25) case showed that the 1q breakpoint lies between BACs RP4-723P6 (chr 1: 216,265,171-216,278,164 GRCh37) and RP11-239I22 (chr 1: 216,441,980-216,535,735 GRCh37) (Figure 2). The *USH2A* gene is therefore directly interrupted. The only other gene in the region encodes the orphan nuclear hormone receptor, ESRRG. The 2p breakpoint is flanked by BACs RP11-410L9 (chr 2: 2,912,073-3,034,544 GRCh37) and RP11-568H24 (chr 2: 3,636,396-3,769,755 GRCh37). Thus, the molecularly-corrected karyotype is 46,XY, t(1;2)(q41;p25.3).

**Figure 3:**
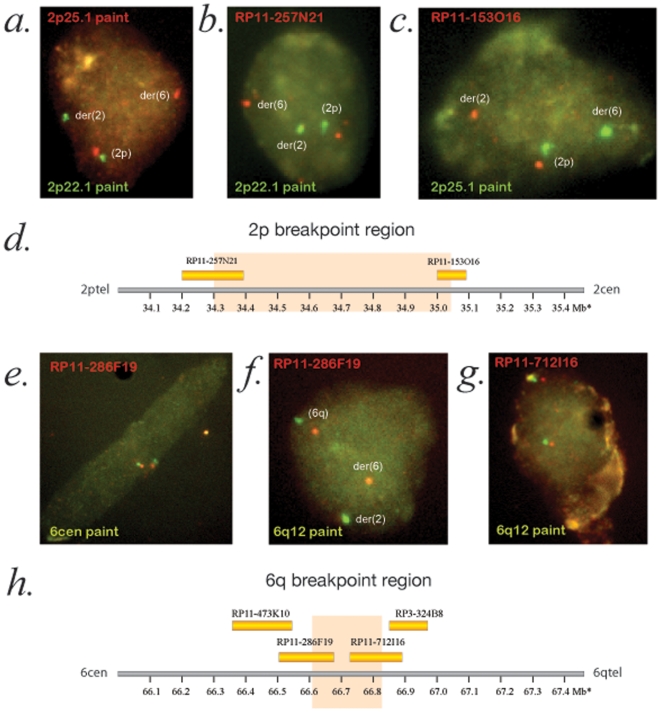
Mapping of the t(2;6) breakpoints using interphase FISH analysis. A. Cohybridization of mini-paints (groups of adjacently mapping BACs hybridized together) for 2p25.1 and 2p22.1 demonstrating that the normal (2p) and derivative chromosomes (der(2) and der(6)) can be clearly distinguished on interphase mapping. B and C. Representative images showing mapping of the clones flanking the 2p breakpoint with RP11-257N21 mapping to the der(6) and RP11-153O16 to the der(2) when co-hybridized with the 2p22.1 and 2p25.1 paint respectively, which are on opposite sides of the breakpoint. D. Diagrammatic representation of the 2p breakpoint region with the orange boxed region containing the breakpoint. There are no known protein-coding genes in this region of DNA. E and F. Mapping of the 6q breakpoint showing the centromeric flanking clone, RP11-286F19, showing colocalization with the centromeric probe (6cen) and the absence of colocalization with the 6q12 paint (which is telomeric to the breakpoint) on one allele. G. Colocalization of the telomeric flanking clone, RP11-712I16, with the 6q12 paint. H. Diagrammatic representation of the 6q genomic region with the orange box representing the location of the translocation breakpoint. This region contains no known protein coding genes.

The breakpoints in the t(2;6)(?p23;?q14) were mapped and the 2p22 breakpoint was found to lie between RP11-257N21 (chr 2: 34,357,572-34,549,712 GRCh37) and RP11-153O16 (chr 2: 35,167,661-35,257,709 GRCh37), and the 6q breakpoint between RP11-286F19 (chr 6: 66,563,349-66,734,396 GRCh37) and RP11-712I16 (chr 6: 66,749,259-66,912,466 GRCh37) (Figure 3). Thus, the corrected karyotype is 46,XY, t(2;6)(p22.3;q12). Neither breakpoint disrupts a known gene or lies within 200 kb of one. Analysis of both regions using ANCORA (http://ancora.genereg.net/) revealed no evidence of clustering of conserved non-coding regions, as is commonly seen in gene desert regions showing long-range *cis*-regulatory control. A full list of the clones used to map all breakpoints is available in Table S2.

### Expression Analysis of Breakpoint Genes

**Figure 4:**
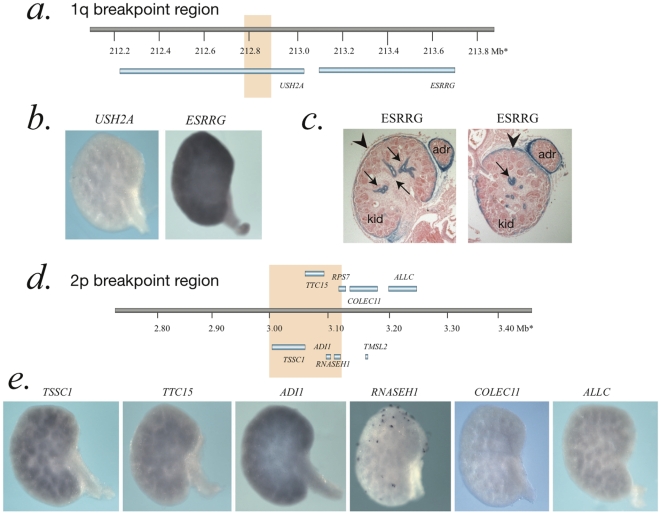
Wholemount *in-situ* hybridization (WISH) of breakpoint genes on embryonic mouse kidneys. A. Diagram of the 1q genomic region with orange box indicating the breakpoint region. B. WISH of the mouse orthologs of the 1q breakpoint genes with representative kidneys from 14.5dpc mouse embryos showing undetectable expression of *Ush2a* and strong expression of *Esrrg*. C. Immunohistochemical analysis of Esrrg in 14.5dpc mouse kidney sections. Strong staining is seen in the collecting ducts (arrows) with weaker staining in the mesothelial capsule (arrow heads) of the kidney (kid) and adrenal gland (adr). D. Diagram of 2p genomic region with orange box indicating the breakpoint region. E. WISH of the mouse orthologs of the 2p breakpoint genes with representative kidneys from 14.5dpc mouse embryos showing undetectable expression of *Colec11* and *Rnaseh1*, intermediate expression of *Tssc1, Ttc15 & Allc* and stronger expression of *Adi1*. No satisfactory probe template could be amplified for *Tmsl2* or *Rps7*.

Wholemount *in-situ* hybridization (WISH) was performed on 14.5dpc embryonic mouse kidneys using riboprobes designed to detect expression of the mouse orthologs of the genes at the t(1;2) breakpoints. In the 1p BP region, no expression of the gene disrupted by the breakpoint, *Ush2a*, was detectable but the neighboring gene, *Esrrg*, was strongly expressed (Figure 4). Immunohistochemical staining of sections of 14.5dpc embryonic mouse kidneys using an antibody against Esrrg showed expression in the future collecting ducts and kidney capsule. WISH expression analysis of six of the genes from the 2p breakpoint region showed intermediate-strong expression of *Adi1*, intermediate levels of expression in *Tssc1*, *Ttc15* and *Allc*, and no detectable expression of *Rnaseh1* or *Collec11*.

## Discussion

An important and consistent finding in this study and others [1], [11] is that renal agenesis, renal hypoplasia, dysplastic and cystic dysplastic kidneys can co-occur in individuals with bilateral lethal disorders of kidney development. This suggests that these apparently distinct clinical endpoints may result from identical or related developmental pathological processes. It is also clear that, although BRADH does occur as an isolated malformation, in the majority of cases it is part of a more general developmental disturbance i.e. the causative genetic or environmental teratogens affect pathways that are critical to both renal and extrarenal development. The scientific rationale for studying patterns of associated abnormalities is to provide clues to specific cellular or morphogenetic processes that may aid the search for causative factors of BRADH. Previous studies have indeed highlighted specific extrarenal malformations that appear to be non-randomly associated with BRAHD, including skeletal anomalies (hemivertebrae, sacral agenesis [36], sirenomelia [37], radial aplasia [38]), cloacal anomalies (imperforate anus with rectovaginal fistula [39], [40]) and Müllerian defects (partial atresia of the vagina, fallopian tubes and uterus in female cases) [41]). An overlap has been noted with the well-known malformation associations: VACTERL (vertebral malformation, anal atresia, cardiovascular anomalies, tracheo-esophageal fistula, esophageal atresia, renal anomalies, limb malformations [OMIM 192350]) [30], MURCS association (Müllerian duct aplasia, unilateral renal agenesis, and cervicothoracic somite anomalies [OMIM 601076]), hemifacial microsomia ([OMIM 164210]) [33], [34] and caudal dysgenesis spectrum (sacral agenesis, sirenomelia [OMIM 600145]) [37].

Our case series supports each of these reported associations but none of these help in elucidating the underlying developmental pathologies. The most convincing human genetic current evidence implicating a specific pathway is the finding of *RET* mutations in one third of the BRAHD cases in a single cohort [19]. In the same study, no convincing mutations were found in genes encoding either the RET ligand (*GDNF*) or its co-receptor, *GFRA*. No associated abnormalities were reported in the cases with *RET* mutations, although mutations in this gene have been associated with another malformation, Hirschprung disease (OMIM 142623) [42]. GDNF/RET signaling is obviously critical for aspects of neural crest and ureteric bud function but, frustratingly, its relationship to other developmental signaling cascades is not yet clear. Apart from RET signaling, there is interesting but relatively weak human genetic evidence implicating Wnt signaling in the control of planar cell polarity. A heterozygous mutation in *WNT4*, encoding a signaling molecule that is thought to repress tissue-specific androgen production in female embryos, has been reported in a female with Müllerian abnormalities associated with unilateral renal agenesis [43]. This is supported by *in vitro* work showing that siRNA knock-down of *Wnt4* in cultured embryonic mouse kidneys has a specific effect of blocking nephron development [44]. Mutations in *VANGL1*, a negative regulator of planar cell polarity/Wnt-signaling [45], have been identified in a case with caudal dysgenesis [46]. This association is supported by studies of the *Isl1*-transgenic mouse model of caudal dysgenesis, which has been shown to have abnormal embryonic expression of several genes encoding components of the Wnt-signaling pathway. How, or if, WNT4 and VANGL1 interact *in vivo* is yet to be defined.

It can be seen from the above data that very large gaps remain in our knowledge of the embryopathology that results in human BRAHD. The motivation for the present study was to find clinical and cytogenetic clues that may identify other molecular players in this disease. Probably the most interesting result was the previously unrecognized finding that one third of cases in our cohort had one or more extrarenal malformations, indicative of a disorder in laterality determination. Disorders of laterality can be divided into complete reversal of normal visceral situs (situs inversus) or partial reversal - left isomerism, right isomerism or heterotaxy. Situs inversus is strongly associated with primary ciliary dyskinesias [47], [48] and there is also a well known association between ciliopathies and renal cystic disease[49]. Heterotaxy is characterized by specific malformations of abdominal structures (malrotation of the gut, polysplenia, midline liver etc.), early heart development (doubling or loss of normally asymmetrical structures) and the lungs (abnormal lobulation). The strong association of these malformation categories with BRADH suggests that they may share a common etiology. This is particularly interesting given the very striking excess of male cases in this group because mutations in the gene encoding the zinc finger transcription factor, ZIC3, have been shown to segregate with cardiac and abdominal heterotaxy as an X-linked recessive trait [50]. Whilst there are no previous reports of renal agenesis associated with this gene, it is interesting to note that one affected male in the original report had renal hypoplasia. Other genes and pathways have been implicated in human heterotaxy, for example intragenic mutations have been identified in *CFC* [51] and *ACVR2B* [52], which are both components of a transforming growth factor-beta signaling system.

On reviewing the available cytogenetic results for our BRAHD cohort we identified one male case with a *de novo*, apparently balanced reciprocal translocation 46,XY,t(2;6)(p22.3;q12). This case had a severe phenotype with multiple malformations associated with BRAHD including sirenomelia and cardiac, abdominal and pulmonary evidence of heterotaxy. We mapped this translocation to determine if either breakpoint disrupted a gene that could be a plausible candidate for the associated phenotype. However, both breakpoints mapped to gene-free regions. There are several possible explanations for this finding: First, the translocation could be coincidental - the fact that the mother of this fetus had insulin-dependent diabetes mellitus, a known risk factor for BRAHD [24], may make this more likely; Second, one or both of the breakpoint regions may be critical for the *cis*-regulation of one or more neighboring genes, although evidence against this comes from the lack of clustering of conserved non-coding regions in these regions or chromatin evidence of enhancer activity in publicly available datasets; Third, the causative mutation may be a deletion in *cis* with one of the breakpoints, which is a relatively common finding in *de novo* translocations [53]. However, we were unable to perform an array-based comparative genomic hybridization on this case as there was no high quality genomic DNA available.

We also report the mapping of another *de novo* translocation in an unrelated male case of isolated BRAHD. We reported clinical and cytogenetic details of this case some years earlier [35]. The 1q41 breakpoint was found to disrupt *USH2A*, a gene that is mutated in the autosomal recessive disorder Usher syndrome 2A (OMIM 276901). This gene was not a good candidate for BRAHD as it was not expressed in the embryonic kidney and homozygous or compound heterozygous loss-of-function mutations do not cause a renal phenotype in humans or knockout mice (Dominic Cosgrove, personal communication). However, we found that the mouse ortholog of the neighboring gene, *ESRRG*, was strongly expressed in mouse embryonic kidneys. Immunohistochemical analysis shows that Esrrg localizes to developing collecting ducts in the 14.5dpc embryonic kidney. *ESRRG*, therefore, seemed to be a reasonable candidate gene for BRAHD. However, targeted inactivation of *Esrrg* in mouse [54] revealed that most homozygous mice die in the first week of life with heart failure, and that no renal phenotype has been noted in these animals. At the 2p25.3 breakpoint several genes could be disrupted or deleted. Expression analyses in embryonic mouse kidneys showed that only *Adi1* was moderately highly expressed. *ADI1* encodes a protein with homology to bacterial aci-reductone dioxygenase, which functions in the methionine recycling pathway. ADI1 localizes to both the cytoplasm and nucleus and has been implicated in both mRNA processing and induction of apoptosis [55], [56]. No mouse knockout for *Adi1* is currently available but it is a potential candidate gene for BRAHD.

This clinical and cytogenetic review of a large cohort of BRAHD cases from a single center has confirmed much of the previously reported clinical associations. In addition, we have identified a strong association of BRAHD with heterotaxy, which implicates a series of signaling pathways and transcription factors that are involved in establishing laterality. The translocation mapping in a case of isolated BRAHD has yielded two plausible candidate genes that merit further investigation. We plan to continue to examine *ESRRG* as a candidate gene by performing functional analyses in embryonic cultured kidneys and assessing renal development in the “knock-out” mouse. We hope that our study will stimulate interest in this common lethal human spectrum of malformations.

## Supporting Information

Table S1 Full list of associated malformations in BRAHD cases.
(0.12 MB DOC)

Table S2 Mapping details of all BAC probes used for Interphase FISH.
(0.13 MB DOC)
