# Supplementary material for: Bilateral Renal Agenesis/Hypoplasia/Dysplasia (BRAHD): Postmortem Analysis of 45 Cases with Breakpoint Mapping of Two De Novo Translocations
Source: PLoS One. 2010 Aug 25;5(8):e12375. doi: 10.1371/journal.pone.0012375 (PMC2928268; doi:10.1371/journal.pone.0012375)
Supplement: Table S1 — Full list of associated malformations in BRAHD cases. (0.12 MB DOC) [file pone.0012375.s001.doc]

**Supplementary Tables**

Table S1: Full List of Associated Malformations

| Malformation | Total |
| --- | --- |
| Undescended testes | 15 |
| Anorectal Atresia | 13 |
| Incomplete lobation right lung | 7 |
| Malrotation | 7 |
| Radial aplasia | 7 |
| Thoracic vertebral malformation | 7 |
| Cleft palate | 5 |
| Micrognathia | 5 |
| Cerebellar hypoplasia | 4 |
| Cleft lip | 4 |
| Meckel Diverticulum | 4 |
| Oesophageal Atresia | 4 |
| Persistent Left Superior Vena Cava | 5 |
| Ventricular Septal Defect | 4 |
| Bicornuate uterus | 3 |
| Transposition of the Great Arteries | 3 |
| Tracheo-Oesophageal Fistula | 3 |
| 13 pairs of ribs | 2 |
| Absent penis | 2 |
| Colonic atresia | 2 |
| Ductal Plate Malformation | 2 |
| Hemivertebrae | 2 |
| Lumbosacral vertebral malformations | 2 |
| Malformation of olivary nucleus | 2 |
| Postaxial polydactyly of upper limbs | 2 |
| Preaxial polydactyly of lower limbs | 2 |
| Rectovesicular fistula | 2 |
| Sacral agenesis | 2 |
| Urethral atresia | 2 |
| 10 pairs of ribs | 1 |
| Aberrant pulmonary artery on right | 1 |
| Absent fallopian tubes | 1 |
| Absent left fibula | 1 |
| Absent nose | 1 |
| Absent testis | 1 |
| Absent upper part of vagina | 1 |
| Absent uterus | 1 |
| Annular Pancreas | 1 |
| Anophthalmia | 1 |
| Atrial Septal Defect | 1 |
| Atresia distal aortic arch | 1 |
| Bicuspid pulmonary valve | 1 |
| Bifid scrotum | 1 |
| Bile duct hamartomas (von Mayenburg complexes) | 1 |
| Craniorachischisis | 1 |
| Double outlet right ventricle | 1 |
| Exomphalos | 1 |
| Facial asymmetry | 1 |
| Facial dysostosis involving maxilla and mandible | 1 |
| Focal cortical hypoplasia | 1 |
| Holoprosencephaly | 1 |
| Hypoplastic lower limbs | 1 |
| Hypoplastic pelvis | 1 |
| Irregular longbone metaphyses | 1 |
| Kyphosis | 1 |
| Left leg amelia | 1 |
| Left leg peromelia | 1 |
| Malformation of dentate nucleus | 1 |
| Megaurethra | 1 |
| Microtia | 1 |
| Mitral stenosis | 1 |
| Multiple Small Bowel Atresias | 1 |
| Occipital encephalocele | 1 |
| Pancreatic Dysplasia | 1 |
| Polymicrogyria | 1 |
| Postaxial polydactyly of lower limbs | 1 |
| Pulmonary artery atresia | 1 |
| Rectovaginal fistula | 1 |
| Right leg dysplastic | 1 |
| Right upper limb phocomelia | 1 |
| Septate upper vagina | 1 |
| Single digit left foot | 1 |
| Sirenomelia | 1 |
| Soft tissue syndactyly upper and lower limbs | 1 |
| Syndactyly | 1 |
| Tetralogy of Fallot | 1 |
| Truncus arteriosus | 1 |
| Underdeveloped diaphragm | 1 |
| Urethral obstruction | 1 |
| Vaginal atresia | 1 |
