# Supplementary material for: Bilateral Renal Agenesis/Hypoplasia/Dysplasia (BRAHD): Postmortem Analysis of 45 Cases with Breakpoint Mapping of Two De Novo Translocations
Source: PLoS One. 2010 Aug 25;5(8):e12375. doi: 10.1371/journal.pone.0012375 (PMC2928268; doi:10.1371/journal.pone.0012375)
Supplement: Table S2 — Mapping details of all BAC probes used for Interphase FISH. (0.13 MB DOC) [file pone.0012375.s002.doc]

Table S2: Mapping details of all BAC probes used for Interphase FISH

| Case | Clone Name | Chromosome Band | Genomic co-ordinates (Ensembl GRCh37) | Position relative to breakpoint |
| --- | --- | --- | --- | --- |
| t(2;6) | RP11-560C7 | 2p24.1 | 23145063-23327513 | Distal |
|  | RP11-404P12 | 2p23.3 | 25843463-26015355 | Distal |
|  | RP11-373D23 | 2p23.2 | 28560953-28718617 | Distal |
|  | RP11-23B13 | 2p23.1 | 30813077-30978722 | Distal |
|  | RP11-559D11 | 2p22.3 | 32652266-32794423 | Distal |
|  | RP11-258F20 | 2p22.3 | 33174879-33350934 | Distal |
|  | RP11-257N21 | 2p22.3 | 34357572-34549712 | Distal |
|  | RP11-153O16 | 2p22.3 | 35167661-35257709 | Proximal |
|  | RP11-288C18 | 2p22.2 | 37051500-37131846 | Proximal |
|  | RP11-278G12 | 2p22.2 | 37798652-37984824 | Proximal |
|  | RP11-173C1 | 2p22.1 | 39153764-39334208 | Proximal |
|  |  |  |  |  |
|  | RP11-506N21 | 6q11.1 | 62581143-62667340 | Proximal |
|  | RP11-767J14 | 6q12 | 63707642-63856093 | Proximal |
|  | RP11-349P19 | 6q12 | 65101827-65152058 | Proximal |
|  | RP1-40C9 | 6q12 | 65783351-65805695 | Proximal |
|  | RP11-722H01 | 6q12 | 66093312-66275577 | Proximal |
|  | RP5-1000G17 | 6q12 | 66240922-66336251 | Proximal |
|  | RP11-473K10 | 6q12 | 66370528-66556850 | Proximal |
|  | RP11-286F19 | 6q12 | 66563349-66734396 | Proximal |
|  | RP11-712I16 | 6q12 | 66749259-66912466 | Distal |
|  | RP3-324B8 | 6q12 | 66844441-66964875 | Distal |
|  | RP11-409K15 | 6q12 | 68158441-68262097 | Distal |
|  |  |  |  |  |
| t(1;2) | RP11-277C14 | 1q24.3 | 171863204-172019044 | Proximal |
|  | RP11-480I12 | 1q32.1 | 202724207-202859974 | Proximal |
|  | RP11-739N20 | 1q32.1 | 204319171-204484249 | Proximal |
|  | RP11-534L20 | 1q32 | 206641696-206747384 | Proximal |
|  | RP11-323K10 | 1q41 | 215075551-215164831 | Proximal |
|  | RP11-438G15 | 1q41 | 215621444-215691989 | Proximal |
|  | RP11-415H9 | 1q41 | 215937175-216006703 | Proximal |
|  | RP5-861H2 | 1q41 | 216083690-216110178 | Proximal |
|  | RP11-22M7 | 1q41 | 216110179-216265170 | Proximal |
|  | RP4-723P6 | 1q41 | 216265171-216278164 | Proximal |
|  | RP11-239I22 | 1q41 | 216441980-216535735 | Distal |
|  | RP11-152K20 | 1q41 | 216535736-216689340 | Distal |
|  | RP11-23B9 | 1q41 | 216689341-216744657 | Distal |
|  | RP11-426K17 | 1q41 | 216744658-216822334 | Distal |
|  | RP11-66M7 | 1q41 | 217145318-217307243 | Distal |
|  | RP11-224O19 | 1q41 | 218469745-218638420 | Distal |
|  | RP11-392O17 | 1q41 | 219415235-219588287 | Distal |
|  | RP11-332J14 | 1q41 | 220691554-220799403 | Distal |
|  | RP11-239E10 | 1q41 | 223288310-223467775 | Distal |
|  | RP11-99J16 | 1q42.2 | 230892110-231089182 | Distal |
|  | RP11-528D17 | 1q42.2 | 233636141-233747560 | Distal |
|  | CTB-160H23 | 1q44 | 249064112-249196418 | Distal |
|  |  |  |  |  |
|  | GS1-8L3 | 2p25.3 | 341000-439000 | Distal |
|  | RP11-168K7 | 2p25.3 | 1633836-1804280 | Distal |
|  | RP11-352J11 | 2p25.3 | 2271126-2411010 | Distal |
|  | RP11-141G5 | 2p25.3 | 2370721-2533998 | Distal |
|  | RP11-744D24 | 2p25.3 | 2411011-2455319 | Distal |
|  | RP11-163G21 | 2p25.3 | 2760668-2912072 | Distal |
|  | RP11-410L9 | 2p25.3 | 2912073-3034544 | Distal |
|  | RP13-512J5 | 2p25.3 | 3579313-3635395 | Fail |
|  | RP11-568H24 | 2p25.3 | 3635396-3770755 | Proximal |
|  | RP11-327H5 | 2p25.3 | 3944642-4148074 | Proximal |
|  | RP13-868N24 | 2p25.2 | 5173307-5209875 | Proximal |
|  | RP11-350H23 | 2p25.2 | 5847931-6037722 | Proximal |
|  | RP11-485O17 | 2p25.2 | 6730817-6821043 | Proximal |
|  | RP11-214N9 | 2p25.1 | 9670825-9767659 | Proximal |
|  | RP11-333O1 | 2p24.3 | 12783039-12986556 | Proximal |
|  |  |  |  |  |
| Probes used for mini-paints: |  |  |  |  |
| 2p25 paint | RP11-434B12 | 2p25.1 | 8689486-8862918 |  |
|  | RP11-327F6 | 2p25.1 | 8863919-9077277 |  |
|  | RP11-785D16 | 2p25.1 | 9077278-9219829 |  |
|  | RP11-734K21 | 2p25.1 | 9219830-9409657 |  |
|  | RP11-385J23 | 2p25.1 | 9409658-9493068 |  |
|  | RP11-400L8 | 2p25.1 | 9493069-9662721 |  |
| 2p22.1 paint | RP11-395B14 | 2p22.1 | 39685766-39835921 |  |
|  | RP11-288L6 | 2p22.1 | 39837916-39987189 |  |
|  | RP11-454P5 | 2p22.1 | 39987190-40212688 |  |
|  | RP11-509C20 | 2p22.1 | 40212689-40348905 |  |
|  | RP11-3F16 | 2p22.1 | 40348906-40491200 |  |
|  | RP11-457F14 | 2p22.1 | 40493195-40670537 |  |
| 6q12 paint | RP1-46B1 | 6q12 | 69382068-69540171 |  |
|  | RP11-258G21 | 6q12 | 69599257-69765699 |  |
|  | RP11-22A15 | 6q12 | 69725331-69881359 |  |
|  | RP1-160B9 | 6q12 | 69881360-69950781 |  |
